# Two proteins, one goal: ELISAs based on p32 and L1R for LSDV antibodies detection

**DOI:** 10.3389/fvets.2025.1695369

**Published:** 2025-10-28

**Authors:** Stefano Baselli, Manuel Corsa, Arianna Bregoli, Benedetta Zanetti, Anna Castelli, Bernd Hoffmann, Milovan Milovanović, Giulia Pezzoni

**Affiliations:** ^1^Animal Protection and Health – Vesicular Virus and Diagnostic Biotechnology, Istituto Zooprofilattico Sperimentale della Lombardia e dell’Emilia Romagna (IZSLER), Brescia, Italy; ^2^Institute of Diagnostic Virology, Federal Research Institute for Animal Health, Friedrich-Loeffler-Institut, Greifswald, Germany

**Keywords:** lumpy skin disease, ELISA, serological assay, P32, L1R

## Abstract

**Introduction:**

Lumpy skin disease virus (LSDV), a member of the *Capripoxvirus* genus, poses a significant threat to livestock health and productivity in both endemic and newly affected regions. The disease is primarily transmitted by blood-feeding insects, leading to fever, cutaneous nodules, lymphadenopathy, and substantial economic losses. While vaccination remains the cornerstone of control efforts, effective surveillance—especially in high-risk areas—relies on robust and scalable diagnostic tools. Although the virus neutralization test is considered the reference standard among serological assays for detecting neutralising antibodies, it is labor-intensive and requires high-containment laboratories.

**Methods:**

In this study, we produced and evaluated two recombinant LSDV antigens: ORF074 (p32), a well-known immunodominant protein, and ORF060 (homologous to the Vaccinia virus L1R), a myristoylated membrane protein identified as a promising immunogenic target. Both proteins were expressed in *E. coli*. Recombinant p32 was purified under native conditions, whereas recombinant L1R required denaturation and refolding. The antigens were used to develop two indirect ELISAs, and were evaluated using sera from experimentally infected cattle, as well as both vaccinated and infected field samples from Albania and Serbia.

**Results and discussion:**

Both assays demonstrated high immunoreactivity and strong concordance with the VNT. These results support the suitability of both antigens for use in serological assays and suggest that a combined, multi-target ELISA approach could enhance diagnostic sensitivity. Once validated for routine use, these novel tools may significantly improve large-scale, cost-effective serological surveillance of LSDV in endemic and at-risk regions.

## Introduction

The *Capripoxvirus* (CaPV) genus, part of the Chordopoxvirinae subfamily within the Poxviridae family, includes the lumpy skin disease virus (LSDV), goatpox virus (GTPV), and sheeppox virus (SPPV), three significant pathogens that specifically infect cattle, goats, and sheep, respectively. They share a sequence homology higher than 90% (1). Lumpy skin disease (LSD) is a serious and highly contagious viral disease primarily affecting cattle and domestic buffalo, but with a potential to infect also other species such as gazelles, camels, yak, etc. (2–4). It is characterized by several clinical signs like skin nodules, enlarged superficial lymph nodes, and occasionally, mortality (5). The symptoms range from mild to severe (6) depending on several factors like the host age and its immunity state, the strain of LSDV, the inoculation route (7), etc. Similarly, SPPV and GTPV cause fever and lesions on the skin and internal organs, with a high mortality rate, particularly in young animals. These diseases are classified by the World Organisation for Animal Health (WOAH) as reportable diseases due to their potential for rapid spread and considerable economic impact in endemic regions. Since 2012, LSD has progressively expanded beyond its traditional range, spreading to the Middle East, Turkey, Iraq, and Iran. In recent years, it has reached Europe and East Asia, causing severe economic losses and triggering large-scale vaccination campaigns to curb its impact and contain further spread (8). The epidemiological scenario has further complicated with the emergence of recombinant LSDV strains. Since 2017, vaccine-like recombinants have circulated in Russia and Asia, arising from poorly regulated vaccine production that enabled recombination between live-attenuated Neethling vaccine strains and field isolates (9). The dominant recombinant lineage, designated cluster 2.5, has become established throughout Southeast Asia and continues to spread across the region (10, 11), affecting countries such as China, Mongolia, Vietnam, Cambodia, Laos, Thailand, Malaysia, Singapore, and Indonesia. In contrast, the Indian subcontinent (including Bangladesh, India, Nepal, Myanmar, Sri Lanka, Pakistan, and Afghanistan) remains primarily affected by Kenyan-like LSDV strains rather than recombinant variants (12). Overall, the continuing spread of LSD across Asia has escalated into a major threat to livestock industries in affected regions, causing significant adverse economic impacts and posing multiple challenges for farmers and policymakers (13). In October 2023, three outbreaks were reported to WOAH in the Russian Federation. Furthermore, several outbreaks were recently reported to WOAH in Libya, Tunisia, Algeria, and Egypt, where the disease remains endemic. Most recently, in June–July 2025, LSDV has re-emerged in the EU for the first time since 2017, with outbreaks in Sardinia (Italy, 21 June) and Auvergne-Rhône-Alpes (France, 29 June) caused by strains phylogenetically linked to the virulent Nigeria 2018 isolate, prompting emergency vaccination of over 650,000 cattle with Neethling-strain vaccines from the EU bank (14–16). Transmission is primarily facilitated by blood-feeding insect vectors, enabling the disease to spread rapidly over long distances (17). However, other routes of infection include contaminated feed and water as well as direct contact between infected and susceptible animals. This highlights the urgent need for innovative diagnostic and preventive measures. The Poxvirus prototype is the Vaccinia virus (VACV), a smallpox virus that was extensively studied and provided fundamental insights regarding viral replication and the interaction of the virus with the host cells (18). As a member of the Poxvirus family, LSDV shares biological properties with VACV. LSDV primarily elicits a cell-mediated immune response, though the humoral response also significantly contributes in counteracting the virus (19). Therefore, serological methods are also important for monitoring pathogen diffusion. Some of these methods can be highly accurate for identifying the presence of antibodies, such as the virus neutralization test (VNT), which is considered the reference method for detecting neutralising antibodies produced by the host. Unfortunately, it is time-consuming, it requires handling live virus and a laboratory with an appropriate biosafety level. Other tests can replace the VNT offering advantages in sample analysis (20). Enzyme-linked immunosorbent assay (ELISA) enables the simultaneous analysis of multiple samples using minimal laboratory equipment. Several ELISAs have been developed employing inactivated and purified virus (21); however, besides biosafety issues, producing and purifying virus demands specialised laboratory equipment and trained personnel. In recent years, various LSDV recombinant proteins have been produced and used as antigens in indirect ELISAs (21–23), including proteins localised to the outer membrane of the extracellular envelope virion (EEV) form (24, 25). To date, the majority of ELISAs developed for LSDV serology rely on ORF074 (p32) recombinant antigen (26, 27), an immunodominant structural protein homologous to the VACV H3L and conserved across all CaPVs, which localises to the inner envelope of the intracellular mature virion (IMV) form. Despite its high immunoreactivity, an exclusive focus on this protein may overlook other valuable antigenic targets. Recent studies have outlined additional LSDV proteins that exhibit promising immunogenic features (28). One such protein is encoded by ORF060, homologous to the L1R protein in VACV, which is a myristoylated transmembrane protein of 23–29 kDa involved in viral entry and membrane fusion, as well as in the formation of IMV (29, 30). ORF060 has emerged as an immunodominant antigen in LSDV and has been suggested as a promising subunit vaccine candidate due to its ability to elicit neutralising antibodies (31). Notably, despite its immunogenic potential, no current serological test employs this protein as antigen. In this study, we produced and characterised two different immunogenic antigens as recombinant proteins, namely the rp32 and the rLSDV ORF060 (rL1R). These antigens were used for developing two serological indirect ELISAs (iELISAs), which were compared by analysing sera from negative cattle collected in Italy when it was classified as CaPV-free, as well as sera from experimentally infected cattle collected weekly up to 28 days post-infection (dpi). Subsequently, sera from vaccinated and infected animals collected during outbreaks and vaccination campaigns in Serbia and Albania were also analysed. The results presented here underscore the value of such rapid and reliable serological tools, which can support surveillance, enhance field diagnostics and contribute to improve monitoring and control of virus spread.

## Materials and methods

### Production of recombinant proteins

#### Proteins cloning

The LSDV Neethling vaccine strain was provided by the Pirbright Institute (Pirbright, United Kingdom), and was used to infect OA3.Ts (*Ovis aries* testis) cells as previously described (32). The viral DNA extraction was performed using DNeasy Blood and Tissue Kit (Qiagen), following the manufacturer’s instructions. Primers and probes for amplifying the extra-virion coding regions of the LSDV analogues p32 and L1R (see Table 1) were designed based on the GenBank sequence AF409138.1. The plasmid pET-CPD, was chosen as a cloning vector (33). At the C-terminus, each construct included, as a fusion protein, the cysteine protease domain (CPD) of *Vibrio cholerae* MARTX toxin to enhance the protein solubility, along with a polyhistidine tag for the subsequent purification step. The Golden Gate method (34) was used to individually insert the two genes of interest in the pET-CPD. Vector and inserts were amplified using Phusion™ Hot Start II High-Fidelity DNA polymerase (Thermo Fisher Scientific) following the manufacturer’s instructions. The thermal cycling protocol for the vector included an initial denaturation at 98°C for 1 min, followed by 35 cycles of denaturation at 98°C for 10 s, annealing at 68°C for 30 s, and extension at 72°C for 6 min, with a final extension step at 72°C for 10 min.

**Table 1:** List of primers used in cloning *H3L* and *L1R* genes.

Primer	5′-3′ sequence
p22 FOR UNI	AGCTTGCGGTCTCGGTCGACGCATTAGCGGATGGAAAATAC
p22 REV UNI	AGCTTGCGGTCTCGGTTATGTATATCTCCTTCTTAAAGTTAAACAAAATTATTTC
H3L F2 Gg	AGCTTGCGGTCTCGTATGGCACATATTCCATTATAT
H3L R2 Gg	AGCTTGCGGTCTCCCGACTGGATGGGATATATAGTA
L1R F Gg	AGCTTGCGGTCTCGTATGGGAGCAGCCGCAAGTATACA
L1R R Gg	AGCTTGCGGTCTCCCGACTCCGTATCCCGAACTTTGAC
T7F	TTAATACGACTCACTATAGGG
p22 CPD R	CACCAAACGTAGCTTTCCATCCAG

The amplification of the target region was performed using extracted LSDV DNA as a template. The thermal cycling profile included an initial denaturation at 98°C for 30 s, followed by 35 cycles of denaturation at 98°C for 10 s, annealing at 65°C for 30 s, and extension at 72°C for 30 s, with a final extension step at 72°C for 7 min. The PCR product was confirmed by agarose gel electrophoresis, and the amplified fragments were excised from the gel, purified with the NucleoSpin Gel and PCR Clean-up (Macherey-Nagel), quantified, and stored at 4°C. Golden Gate cloning was then performed for each amplified gene in separate reactions. Each 15 μl reaction included a 1:1 molar ratio of the linearized vector and insert, 1.5 μl 10X T4 DNA Ligase Reaction Buffer (NEB), 1.5 μl unacetylated BSA (NEB), 1 μl T4 DNA Ligase and 1 μl BsaI-HF®v2 (NEB). The reaction mixes were incubated at 37°C for 1 h, followed by an incubation at 55°C for 5 min. Then, 5 μl of each ligation product was used to transform *E. coli* One Shot™ Mach1™ T1 Phage-Resistant Chemically Competent cells (Invitrogen) following manufactorer’s instructions. After 24 h of incubation at 37°C, the plates were checked for colonies growth. Colonies screening and selection to obtain the plasmid clone to be used for the protein expression was carried out as previously described (35).

### Proteins expression and purification

Plasmid DNA from clones with confirmed nucleotide sequence was used to transform *E. coli* BL21(DE3) chemically competent cells (NEB) according to the manufacturer’s recommendations. Transformed cells were plated on LB agar supplemented with 100 μg/ml ampicillin to select for resistant recombinant colonies. After O/N incubation at 37°C, a single colony from each construct was inoculated into 25 ml of LB broth containing ampicillin and incubated O/N at 37°C with shaking. The cultures were used to inoculate 1 L of LB broth supplemented with ampicillin (1:200 dilution) in two separate flasks, one for each recombinant protein. Cultures were incubated at 37°C with shaking, and growth was monitored by measuring the optical density at 600 nm (OD_600_) using a DU 730 spectrophotometer (Beckman Coulter). Upon reaching an OD_600_ of 0.5, protein expression was induced by adding isopropyl β-D-1-thiogalactopyranoside (IPTG, Roche) to a final concentration of 1 mM. After 4 h of induction, the whole cultures were centrifuged at 4000 x g for 15 min at 4°C using an Avanti J-26S XP centrifuge (Beckman Coulter). Supernatants were discarded and the resulting cell pellets were stored at −80°C for subsequent analysis.

The rp32 was expressed by resuspending the pellet in 20 ml of lysis buffer (500 mM NaCl, 50 mM TrisHCl, 10% glycerol, pH 7.5) supplemented with protease inhibitors (Complete EDTA—free, Roche). This suspension was frozen at −80°C for 30 min and then rapidly thawed. Subsequently, the sample was kept on ice and sonicated using an MSE Soniprep 150 sonicator (Labexchange). The sonication was performed in 5 consecutive cycles, each lasting 30 s at an amplitude of 14–16 microns. A 10-s pause was included between each cycle to prevent the sample from overheating. Finally, the lysate was centrifuged at 10,000 × *g* for 30 min at 4°C, and the resulting supernatant was collected and stored at −20°C. The described pellet treatment was repeated two times in 15 ml of lysis buffer. The rp32 expression was evaluated in SDS-page carried out as described below. The rL1R protein was expressed by treating the pellet similarly to the rp32 pellet, with the exception of using a denaturing lysis buffer. This treatment yielded three supernatants of approximately 20 ml each, which were combined and subjected to purification and refolding. Renaturation and purification of the rL1R protein were carried out on-column using the Immobilized Metal Affinity Chromatography (IMAC) technique with HisTrap™ HP His-tag protein purification columns and an ӒKTA pure™ chromatography system (Cytiva, Marlborough, MA, USA). Protein refolding was facilitated by an on-column imidazole gradient (15–500 mM), allowing gradual elution and proper folding. The protein was then subjected to O/N dialysis in a buffer containing 25 mM Tris–HCl and 20 mM NaCl at pH 8. The purity and apparent molecular weight of the refolded rL1R protein were confirmed by SDS-PAGE. The concentration of renatured rL1R and the crude cell lysate of rp32 was determined by measuring the UV absorbance at 280 nm on the UV–VIS Agilent 8,453 spectrophotometer (Agilent Technologies, Santa Clara, CA, USA).

The same procedure described above for the production of the proteins of interest was carried out also to obtained a crude cell lysate of the empty plasmid pET-CPD, to be used in ELISA assay as additive in sera dilution buffer to block specific reactions against the CDP fusion protein. The total amount of the protein in the crude cell lysate was measured with BCA assay (Pierce) and found to be 1.48 mg/ml. After quantification, the crude lysate was aliquoted and stored at −20°C until use.

### Recombinant proteins characterisation

The recombinant proteins were evaluated both in Western blotting (WB) and iELISA using specific monoclonal antibodies (mAbs). For WB, a total of 150 ng of each protein preparation was denatured in sodium dodecyl sulphate (SDS) and β-mercaptoethanol and separated onto a precast polyacrylamide gels with 4–12% gradient concentration (NuPAGE™ Bis-Tris Mini Protein Gels). Proteins were then transferred onto polyvinylidene difluoride (PVDF) membranes using Trans-Blot® Turbo™ Transfer System (Bio-Rad Laboratories, Hercules, CA, USA) membranes were blocked O/N at 4°C with 5% skim milk and 0.2% Tween-20 in PBS 1×. After three washes of 10 min each with PBS 1x containing 0.2% Tween-20, the membranes were incubated for 1 h at room temperature (RT) with Penta·His Antibody, BSA-free (QIAGEN, Hilden, Germany) diluted to 0.2 μg/ml in 2% skim milk and 0.2% Tween-20 in PBS 1×, according to the manufacturer’s instructions. Following three additional washes, membranes were incubated with HRP-conjugated goat anti-mouse IgG (1:250 dilution) for 1 h at RT. The membrane was washed and detected with Novex™ HRP Chromogenic Substrate (TMB) (Invitrogen, Carlsbad, CA, USA) after 5-min development period. The rp32 was also evaluated with the p32 specific 2C6 mAb (32) used as the primary antibody.

Four p32-specific (2C6, 2F10, 2F12, and 6B12) and two L1R-specific mAbs (6A12 and 2H10) were used to evaluate the antigenic properties of LSDV recombinant proteins. These mAbs were generated against LSDV antigens as previously described (32). Three out of four mAbs (2C6, 2F10, 2F12) demonstrated reactivity in WB against the viral antigen, revealing a band with a molecular weight corresponding to the p32, while 6B12 and both the L1R mAbs did not show detectable reactivity (data not shown).

Both the proteins and the mAbs were evaluated at different concentration in iELISA. The proteins were directly adsorbed onto microplate wells and 2-fold diluted starting from a concentration of 10 μg/ml, while the starting concentration of the mAbs was 1 μg/ml and then 2-fold diluted, the recognition of the antigen by the mAb was traced by an anti-mouse IgG HRP-conjugated mAb at fixed dilution.

### Sera collection

This study uses sera samples obtained from the Friedrich-Loeffler-Institut (FLI—Greifswald-Insel Riems, Germany) and from national surveillance and routine self-control activities in cattle in Italy. The study did not require approval by an ethics committee because the sera were obtained either from previous studies already covered by ethical approval or from routine diagnostic activities.

Experimental cattle sera were collected during infections conducted at FLI. Specifically, 90 sera samples were obtained from 18 cattle (36), including six animals inoculated with the LSDV Neethling attenuated vaccine strain, eight infected with the LSDV strain circulating in North Macedonia in 2016, and four infected with an LSDV Nigerian strain isolated in 2019 (37). Sera were taken from each animal prior to infection and then weekly for up to 28 dpi.

A total of 332 field serum samples were collected in Serbia in 2017 from cattle vaccinated against LSDV, as previously described (38), and they were kindly provided by FLI. Specifically, 263 of these samples were obtained from 96 animals that had been sampled at multiple time points following a two-dose vaccination with the Neethling strain. These animals were located on farms where no clinical cases of LSD had been reported. Sampling took place prior to the administration of the second vaccine dose (82 samples), 1 month after the second dose (91 samples), and 5 months post-second dose (90 samples). Additionally, 69 sera previously utilised in studies on passive immunity transfer were included in the analysis. These samples were derived from 21 immunised cows and 24 of their calves, collected at birth (day 0) and 2 weeks later (day 14).

In total, 658 cattle sera collected in Albania during the outbreak that occurred in 2016–2017 were tested. This group involved both 358 sera of vaccinated animals (with inactivated LSDV Neethling strain) without any clinical sign and 300 sera of infected animals. All the sera were previously analysed with VNT (39).

Sera of negative animals were provided by several livestock in Lombardy region in Italy (collected in 2022–2023). In total, 421 cattle sera were analyzed both with rp32 and rL1R iELISAs.

All the sera were previously analyzed using VNT that was carried out as described earlier (36). The negative sera were assumed to be negative in VNT.

### Indirect ELISA

To test the panel of sera, two serological iELISAs were set up. Since we were not able to purify the rp32, it was captured using the 2F10 mAb while the rL1R was directly adsorbed onto the microtiter wells. The optimal concentrations of 2F10 mAb, both proteins and the HRP-conjugated anti-bovine IgG 1G10 mAb were determined based on the response (e.g., optical density) to a positive and a negative serum sample diluted 1/50.

The mAb with concentration 3 μg/ml was adsorbed onto microtiter plates (NUNC, Maxisorp, Roskilde, Denmark) in a carbonate/bicarbonate solution (pH 9.6) and incubated O/N at 4°C. After three washing steps with washing buffer (PBS with 0.05% Tween-20), the rp32 protein was added at a saturating concentration (approximately 2 μg/ml) and incubated for 1 h at 37°C in a dilution buffer (PBS with 0.05% Tween-20 and 1% yeast extract). The antigen was applied to odd rows, while even rows were incubated with dilution buffer alone; subsequently, three additional washes were performed. Sera were then diluted 1:50 in a suitable buffer (PBS with 0.05% Tween-20, 0.5% casein sodium, and 50 μg/ml *E. coli* extract with CPD) and added to each well (both in odd and even rows), allowing each serum sample to be analyzed with and without antigen. This was followed by a 1 h incubation at 37°C and three washes with washing buffer. An anti-ruminant IgG mAb (1G10) conjugated with peroxidase (HRP) was diluted in a buffer containing PBS with 0.05% Tween-20, 0.5% casein sodium, was added to each well and incubated for 1 h at 37°C. After three final washes, 0.5 mg/ml of OPD (o-phenylenediamine) diluted in phosphate–citrate buffer (pH 5.6) and supplemented with 0.02% H_2_O_2_ was distributed and incubated for 10 min at RT; the reaction was stopped with 1 M H_2_SO_4_. Plates were read using a Multiscan Ascent spectrophotometer (Multiscan Ascent spectrophotometer, Thermo Fisher Scientific, Waltham, MA, USA) at a wavelength of 492 nm. The net OD value was calculated by subtracting the OD of the well without antigen from the OD of the well containing the antigen.

For the rL1R-based iELISA, the antigen was directly adsorbed onto the plate as a purified antigen at a saturating concentration (5 μg/ml), in a carbonate/bicarbonate solution (pH 9.6) and incubated O/N at 4°C. A few wells without antigen were included to serve as blank control. After three washes with washing buffer, sera were diluted 1:50 in the buffer previously described for p32 and added to each well for a 1 h incubation at 37°C. Subsequently, three washes were performed, and the HRP-conjugated 1G10 mAb was diluted in the same buffer used before, added and incubated for 1 h at 37°C. After three final washing steps, the same OPD reaction explained above was performed. The result for each serum sample was corrected by subtracting the blank.

## Results

### Recombinant proteins expression, purification and characterization

**Figure 1:**
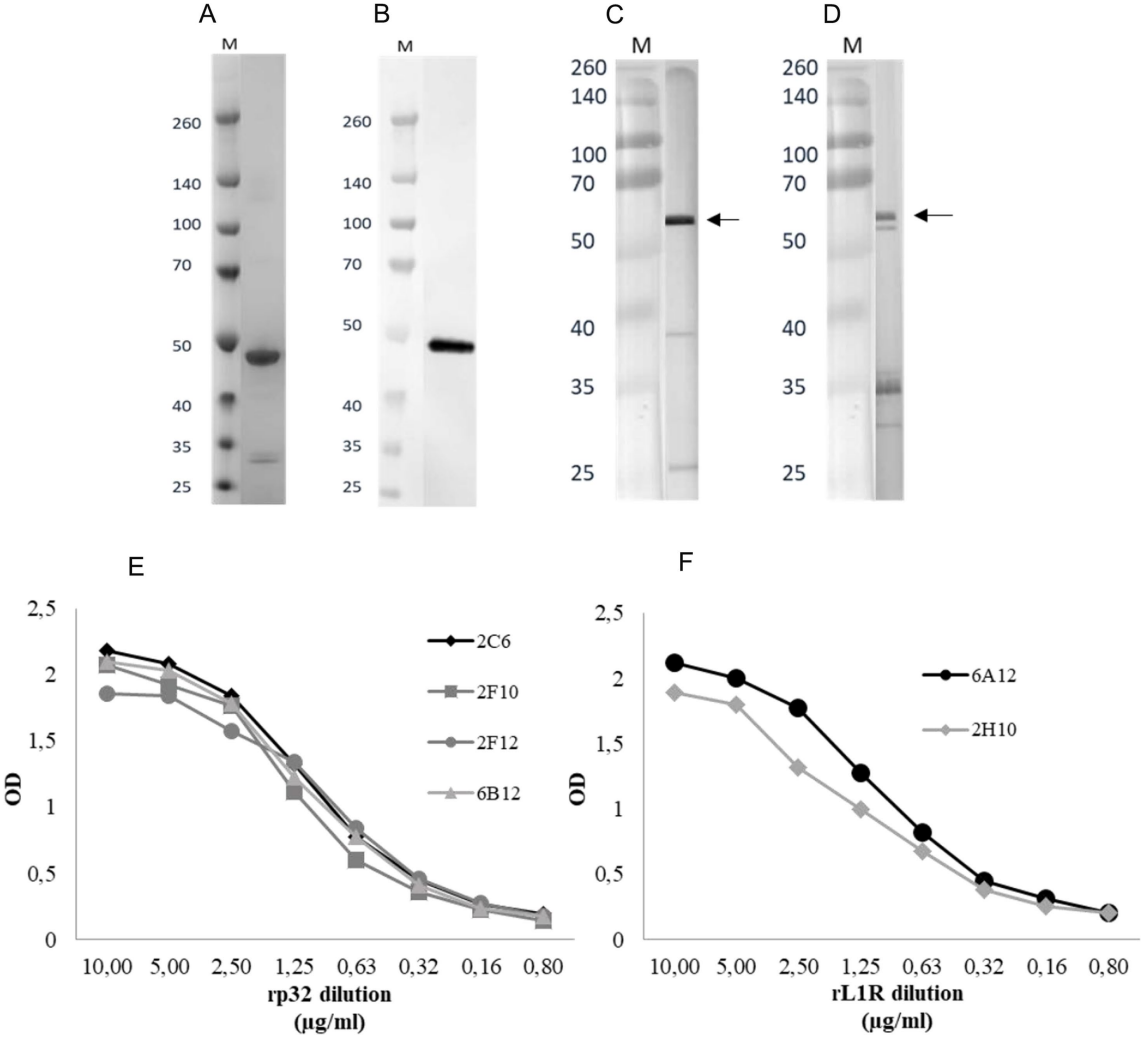
**(A,B)** SDS-PAGE following purification and renaturation of rL1R protein **(A)** and WB of renatured rL1R with Penta·His Antibody as primary antibody **(B).** A total of 150 ng/well of recombinant protein was loaded in the WB. M: molecular weight marker (kDa). **(C,D)** WBs of rp32 crude lysate with Penta·His Antibody **(C)** and 2C6 mAb **(D)** as primary antibodies. The expected molecular weight of the recombinant protein is indicated by an arrow. M: molecular weight marker (kDa). **(E,F)** Titration of rp32 **(E)** and rL1R **(F)** proteins in iELISA with specific mAbs. Proteins were adsorbed onto the plate at a starting concentration of 10 μg/ml and two-fold serially diluted. The mAbs were diluted to obtain a concentration of 1 μg/ml.

The *p32* and *L1R* genes were successfully cloned into two separate pET-CPD vectors (33) using the LSDV Neethling strain. Each construct was individually expressed in 1 L of *E. coli* culture. The pellet was recovered from the rp32 centrifuged culture and processed with native lysis buffer, whereas the rL1R pellet was treated with denaturing lysis buffer. The rL1R protein was purified via Immobilized Metal Affinity Chromatography (IMAC), followed by renaturation step. The purity and molecular weight of rL1R were subsequently confirmed by SDS-PAGE (Figure 1A), revealing a distinct protein band at approximately 50 kDa, consistent with the theoretical molecular weight of 49 kDa when accounting for the 23 kDa CPD tag. Conversely, rp32 protein was not further purified and was retained as crude lysate. The production of the rL1R protein from a 1-liter bacterial culture yielded approximately 2 mg of protein.

Western blot (WB) analysis confirmed the identity of the expressed proteins. As shown in Figures 1B,C, the WB performed using an anti-His mAb detected a band at approximately 50 kDa for rL1R, consistent with its predicted molecular weight. For rp32, a more intense band appeared just above 50 kDa when probed with both the anti-His mAb and the rp32-specific mAb (2C6), aligning with its expected molecular weight of ~55 kDa. Additionally, several lower-molecular-weight bands were observed with both antibodies, likely representing degradation products.

Both proteins reacted in the iELISA with their respective specific mAbs, showing a protein concentration-dependent response (Figures 1E,F).

### Indirect ELISA

**Figure 2:**
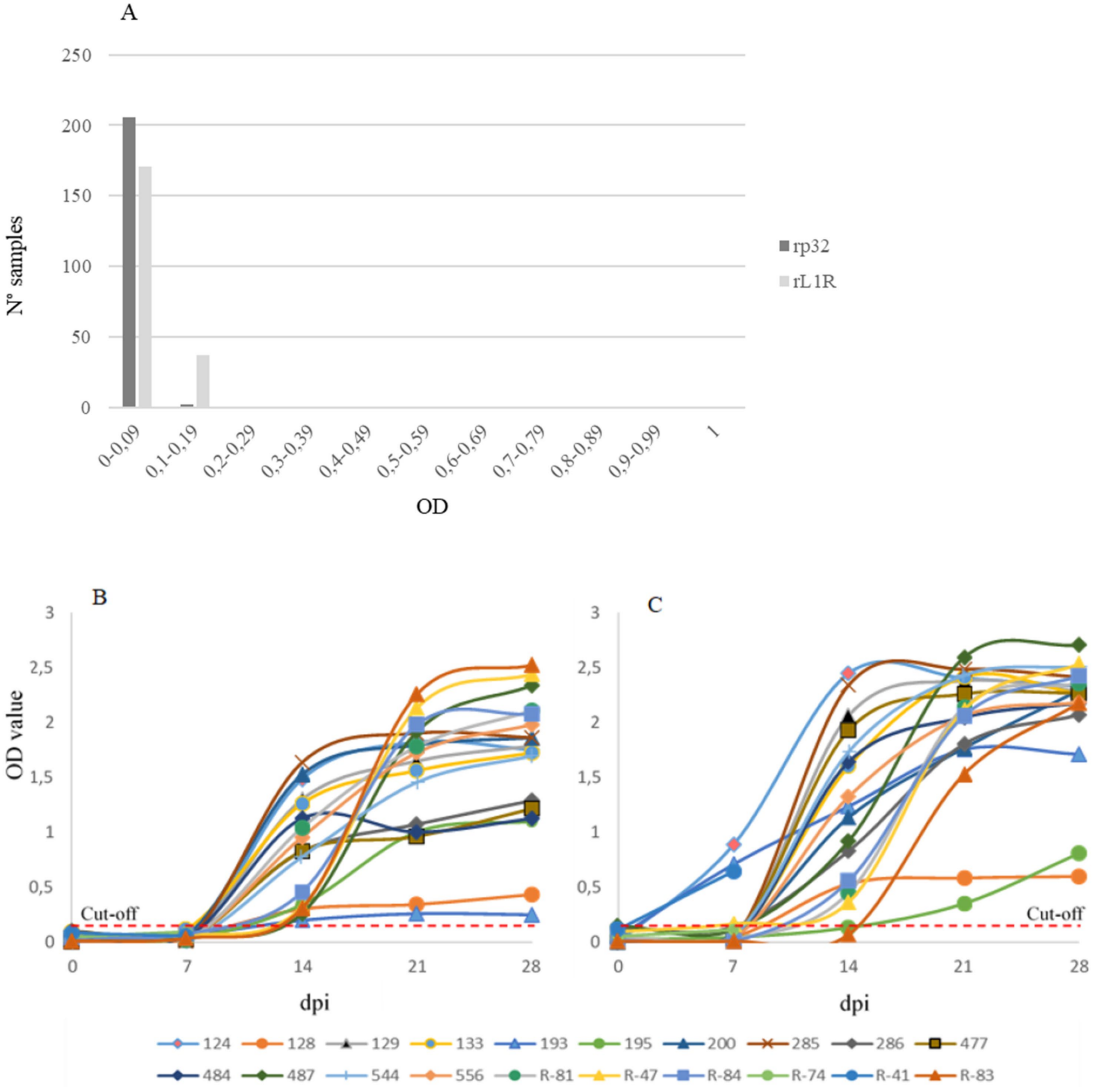
Distribution of negative sera **(A)**, and experimentally infected sera **(B,C)** collected before infection and weekly up to 28 days post infection (dpi). Both iELISAs with rp32 **(B)** and rL1R **(C)** as antigens were evaluated. On the x-axis dpi are shown, while on the y-axis the optical density (OD) value is reported. The red dashed lines indicate the cut-off values for both analyses.

The developed assays were initially evaluated using sera collected from a CaPV-free country at the collection time (negative samples, Figure 2A), alongside sera from experimentally infected cattle sampled before infection and weekly up to 28 days post-infection (dpi, Figures 2B,C). This evaluation aimed to assess the ability of both assays to accurately distinguish between LSDV-positive and negative sera.

Results were expressed as optical density (OD) values. For negative sera, ODs ranged from 0 to 0.113 in the rp32 ELISA and from 0 to 0.197 in the rL1R ELISA. In the rp32 ELISA, all sera collected from experimentally infected cattle at 0 and 7 dpi showed OD values below 0.2 (range 0–0.12), while sera collected from 14 to 28 dpi demonstrated seroconversion with higher OD values (range 0.2–2.53). In the rL1R ELISA, all samples collected at 0 dpi had OD values within the range 0–0.15. At 7 dpi, only three out of 20 cattle sera exhibited OD values between 0.65 and 0.9, while the remaining samples showed values between 0 and 0.17. From 14 dpi onwards, all sera showed increasing OD values, except for two samples, which exhibited a clear seroconversion at a later time point. Considering the distribution of OD values from negative and positive sera, a cut-off value of 0.2 OD was established for both assays.

**Figure 3:**
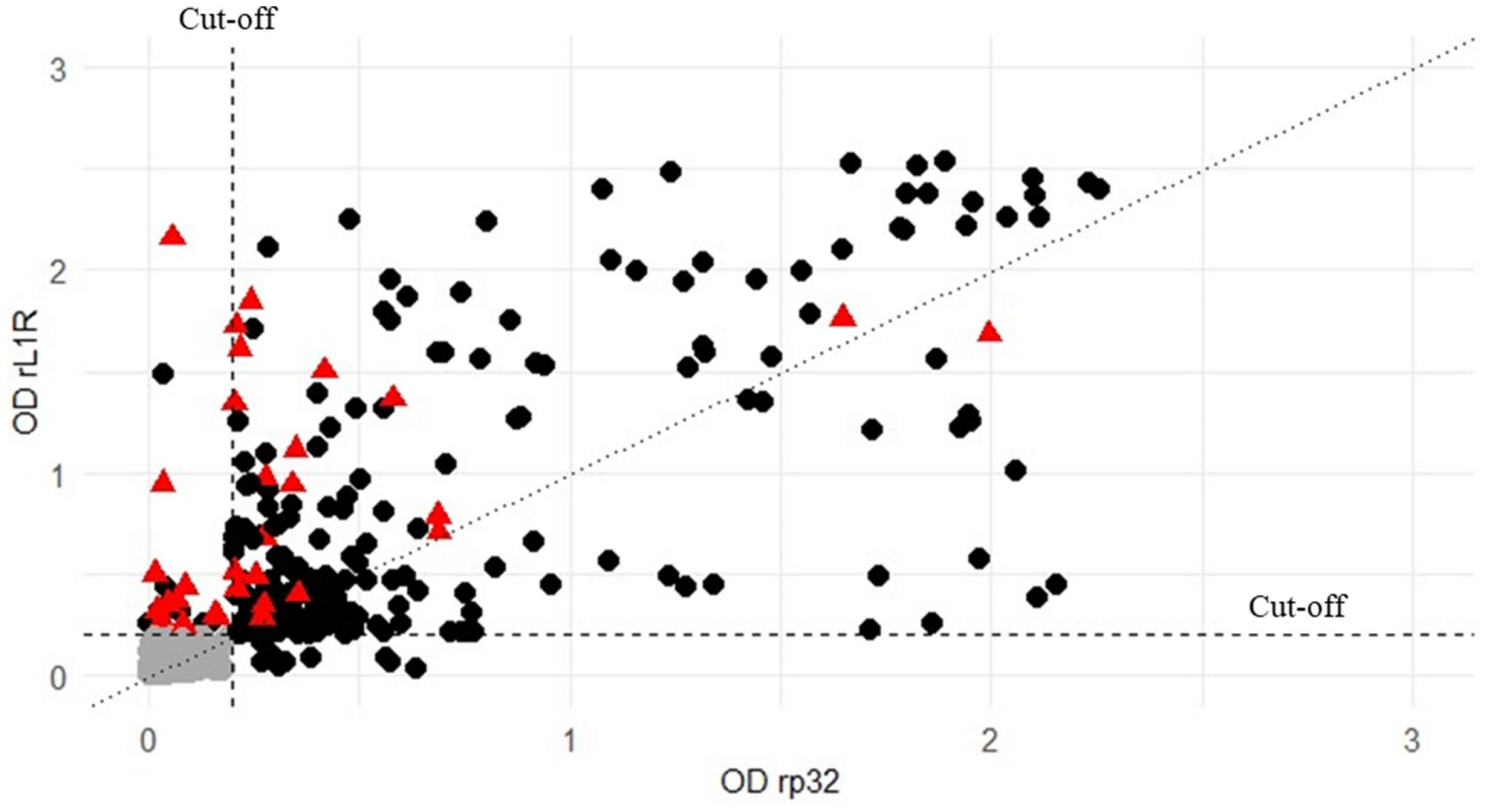
Distribution of all the 658 field sera both analysed with rp32 (x-axis) and rL1R (y-axis) ELISAs and compared to the results obtained by VNT (negative or positive). Values are expressed as OD on both axes. Black circles represent samples positive in VNT, grey circles represent those samples negative in all the tests, while red triangle indicate VNT-negative sera that tested positive in at least one ELISA. The vertical and horizontal dashed lines represent the cut-off for both the analyses.

Serological analysis was performed on field sera collected in Serbia during the vaccination campaign in 2017 and on sera obtained from Albania during the lumpy skin disease outbreak in 2016–2017. All samples were analysed using two different iELISAs methods to assess antibodies presence (Figure 3). The results produced by the two ELISA methods showed a high level of concordance, with 89% of the tested samples yielding consistent results across both ELISAs and VNT (Table 2). The agreement between the two ELISA methods was 92%, including a 3% of the samples negative only in the VNT, with 75% of them (15 out of 20) showing OD values of rL1R ELISA between 0.5 and 1.9. Additionally, 4% of the samples were positive in only one of the two ELISA tests and the VNT, whereas another 4% of the samples were positive in only one test (either ELISA or VNT).

**Table 2:** Field sera of vaccinated cattle collected in Serbia and Albania (vac_sera) and field sera from infected herds in Albania (inf_sera) were analysed using VNT and both rL1R and rp32 iELISAs.

n° pos tests	rL1R_ELISA	rp32_ELISA	VNT	vac_sera (%)	inf_sera (%)	tot_sera (%)
3	+	+	+	152 (42)	58 (19)	210 (32)
0	−	−	−	174 (49)	207 (70)	381 (57)
2	+	+	−	1 (0)	19 (6)	20 (3)
	+	−	+	9 (3)	1 (0)	10 (2)
	−	+	+	8 (2)	2 (1)	10 (2)
1	+	−	−	1 (0)	12 (4)	13 (2)
	−	+	−	0 (0)	1 (0)	1 (0)
	−	−	+	13 (4)	0 (0)	13 (2)
Total				358	300	658

The table presents the distribution of sera in the different results combinations for the three tests. Values percentages were rounded to the nearest whole number for readability.

## Discussion

LSD threatens the livestock industry through long-distance transmission by blood-feeding insects. Although vaccination remains the most effective control method, its success depends on identifying high-risk regions and transmission season, an effort demanding enhanced diagnostic tools. In this framework, ELISAs offer particular advantage among serological tests, delivering results comparable to the gold-standard VNT while enabling rapid, high-throughput screening. Recent advances have strengthened their role as key diagnostic tools for LSD and related Capripoxvirus infections, with different assay formats—such as indirect, competitive, or double-antigen ELISAs—being evaluated against conventional standards (40–42). The growing use of recombinant proteins as antigens, supported by modern expression systems and innovative designs, has further improved diagnostic performance and enabled broader application in surveillance and vaccine monitoring.

In the current scenario, this study aimed at comparing the antigenic properties of two LSDV proteins—ORF 074 (p32) and the ORF 060 product (a homologue of Vaccinia virus H3L and L1R, respectively)—to determine their suitability for the development of LSDV serological ELISA. The p32 protein, a well-established immunogen among CaPVs, has already demonstrated diagnostic value in serological assays targeting GTPV, SPPV, and LSDV (43, 44). In contrast, although the L1R analogue has been less investigated in the context of LSDV, it elicits immunogenic responses in Vaccinia virus, making it a compelling alternative target. The L1R protein is a highly conserved, myristoylated protein essential for viral entry and proper virion assembly (45, 46). Its structural conservation across poxviruses—including the myristoylation motif and six cysteine residues forming disulfide bonds—highlights its critical role in viral infectivity and immune recognition (47). The high degree of conservation, combined with its ability to elicit neutralising antibodies, makes L1R a promising target for diagnostics against CaPVs. Consistently, our analyses indicate that the L1R sequence used here is highly conserved across both historical/vaccine strains (cluster 1) and contemporary recombinant field isolates (cluster 2.x), supporting its applicability for detecting circulating LSDV. Furthermore, aligning the L1R sequence used in this study with sequences from parapoxviruses and other bovine-infecting viruses did not produce any significant matches, suggesting minimal risk of cross-reactivity outside Capripoxviruses. Both proteins were successfully expressed in *E. coli* using a *Vibrio cholerae* CPD tag to enhance solubility. This approach was effective for rp32 that was extracted under native conditions, whereas rL1R required denaturation and refolding, highlighting its more complex folding requirements. Western blot analysis confirmed the successful expression of both proteins, with rp32 showing a strong signal at the expected molecular weight, despite some proteolytic degradation. Importantly, rL1R was detected at the predicted molecular weight following refolding, confirming successful purification and at least partial restoration of its native-like structure. The antigenicity of both the recombinant proteins was assessed by ELISA using a panel of mAbs produced against the viral antigen (data not shown). The rp32 was recognised by mAbs targeting linear epitopes, as confirmed by their reactivity in WB against the viral p32 antigen. Notably, rp32 and rL1R were recognised by conformation-dependent mAb (6B12 and 6A10/2H10, respectively) further supporting the preservation of native structural elements in both proteins. Although the L1R protein required denaturation and refolding—raising the possibility of partial misfolding or reduced stability—its recognition by antibodies specific for conformational epitopes suggests that refolding successfully restored the critical antigenic structure. Two different iELISAs were developed using the produced antigens. In the rp32 ELISA, the antigen was immunopurified with the 2F10 mAb because direct purification of the antigen was not feasible. This mAb had previously proven effective in exposing also the viral antigen for recognition by antibodies in positive sera (32). The rL1R was directly adsorbed onto the microplate wells, as it could be successfully purified and refolded, and none of the L1R-specific mAbs enabled effective capture of the antigen for antibodies recognition in positive sera (data not shown). The two iELISAs were evaluated using a panel of sera, including samples from an experimental infection and 421 sera collected from LSD-negative animals. The optical density (OD) distribution of the negative sera, together with the evaluation of sera from experimentally infected animals, allowed us to establish cut-off value of 0.2 OD for both the tests. In the rp32 iELISA, all sera from the experimental infection seroconverted at 14 dpi. In contrast, while the rL1R iELISA also detected seroconversion in most samples at 14 dpi, three sera showed an earlier response at 7 dpi with high OD values, whereas two sera seroconverted only at 21 dpi. This variable detection pattern suggests that the rL1R iELISA may identify early antibody responses in some cases but may also delay detection in others, when compared to the rp32 assay. The reason for this variability remains unclear and may reflect differences in individual immune responses or antigen-specific kinetics. The tests were further compared by analysing 658 field sera collected from vaccinated and infected animals during outbreaks in Serbia (2017) and Albania (2016–2017). All the sera had been previously tested by VNT. Overall, concordant results between the two in-house iELISAs and the VNT were observed in 89% of the samples, with 11% showing discordant results. Agreement with the VNT increased to 95% when concordant results from either ELISA were considered, suggesting that combining both assays may enhance the overall sensitivity of ELISA-based detection. Notably, 17 out of 20 of these discordant sera were also positive in a previously validated iELISA based on whole-virus antigen (data not shown). Furthermore, 18 of the 20 sera that tested positive in both in-house ELISAs but negative by VNT were also positive in the whole-virus antigen iELISA. This finding suggests that low-titre samples may be misclassified by VNT due to its lower sensitivity during early or weak antibody responses. A total of 27 sera yielded positive results in only one of the assays. Among these, 14 samples were positive in at least one ELISA, but only four had previously tested positive using viral antigen. Conversely, within the group of sera that tested positive exclusively by VNT, only one matched earlier result (data not shown). These discrepancies may also reflect the IgG-specific nature of our ELISAs, which could contribute to divergent outcomes particularly for the samples sampled at the beginning of the infection. Such cases should therefore be interpreted within the broader diagnostic context. Compared to the VNT, which is labor-intensive, requires BSL-3 facilities, and takes several days to perform, the iELISAs presented here can be carried out in BSL-2 laboratories with standard equipment, producing results in few hours. These features, together with reduced costs per sample, make them attractive tools for large-scale surveillance and vaccine monitoring programs.

In conclusion, the rL1R-based iELISA demonstrated comparable performance to the rp32-based assay in distinguishing positive from negative sera, with an overall agreement of 94%. Given their high concordance with VNT, both iELISAs proved to be reliable and effective tools for serological diagnosis. Importantly, rL1R also proved effective in detecting positive sera from experimentally infected goats and sheep, suggesting a potential broader relevance for CpV serology beyond cattle (data not shown). Taken together, these results indicate that both iELISAs are robust diagnostic tools, and that the inclusion of L1R in a combined multi-target approach may substantially strengthen antibody detection and enhance overall diagnostic performance in future applications.

## Data Availability

The data supporting the findings of this study are available within the article.
